# Incidence and Predictors of Left Ventricular Thrombus Formation After Acute Myocardial Infarction With ST-Segment Elevation

**DOI:** 10.7759/cureus.50495

**Published:** 2023-12-14

**Authors:** Devrim Kurt, Emre Yılmaz, Sencer Çamcı, Ertan Aydın, Şükrü Çelik

**Affiliations:** 1 Cardiology, Giresun University Faculty of Medicine, Giresun, TUR; 2 Cardiology, Bursa Postgraduate Hospital, Bursa, TUR; 3 Cardiology, Trabzon Ahi Evran Thoracic and Cardiovascular Surgery Training and Research Hospital, Trabzon, TUR

**Keywords:** transthoracic echocardiography, left ventricular aneurysmatic segment, wall motion score index, st elevated myocardial infarction (stemi), left ventricular thrombus

## Abstract

Background and objective

Our prospective study aimed to evaluate the frequency and risk factors of left ventricular thrombus (LVT) occurring after acute ST-segment elevation myocardial infarction (STEMI) in the era of primary percutaneous coronary intervention (PCI).

Methods

Our study included 131 patients diagnosed with acute STEMI who were followed up and treated. The presence of the thrombus was determined by transthoracic echocardiography (TTE). Study patients were evaluated as cases of thrombus (+) and thrombus (-). The relationship of electrocardiographic measurements such as the number of leads with pathological Q waves, ST segment deviation score, QT dispersion, and echocardiographic measurements such as ejection fraction (EF), end-systolic and end-diastolic volumes, and wall motion score index (WMSI) with LVT was investigated. LVT risk factors were identified.

Results

The median age of the study patients was 59.7 ± 11.7 years, and 84.7% were male. The incidence of LVT was 17.6% (23 patients). While the anterior STEMI rate was 86.9% in the thrombus (+) group, it was 50.9% in the thrombus (-) group (p<0.001). While WMSI was 2.1 ± 0.44 in the thrombus (+) group, it was calculated as 1.40 ± 0.31 in the thrombus (-) group (p<0.001). In the thrombus (+) group, EF was found to be lower, end-systolic and end-diastolic volumes were higher, and the rate of moderate and severe mitral regurgitation and the rate of aneurysmatic segment detection were higher. LVT had a moderate correlation with WMSI (r: 0.613; p<0.001), the presence of an aneurysmatic segment (r: 0.549; p<0.001), and EF (r: -0.514; p<0.001). Presentation with anterior STEMI (odds ratio [OR]: 4.266; p<0.001), WMSI (OR: 7.971; p=0.012), the number of leads with pathological Q waves detected at discharge (OR: 3.651; p=0.009), the presence of an aneurysmatic segment (OR: 2.089, p=0.009), and EF (OR: 1.129, p=0.006) were identified as independent risk factors of the presence of LVT. The area under the curve for WMSI was found to be 0.910 (95% CI: 0.852-0.968). A WMSI cut-off of 1.56 identified LVT with 91% sensitivity and 70% specificity (Youden index: 0.617).

Conclusion

In the primary PCI era, LVT incidence after acute STEMI is still significant. Anterior STEMI, the number of leads with pathological Q waves detected at discharge, WMSI, aneurysm formation, and low EF are independent risk factors for LVT. Among these risk factors, the variable with the highest diagnostic power is WMSI.

## Introduction

ST-segment elevation myocardial infarction (STEMI) is still one of the most lethal diseases today despite all the advances in its treatment. One of the most serious complications of STEMI is left ventricular thrombus (LVT) formation. Even in current studies, the incidence of LVT after STEMI is at significant levels [1]. LVT causes not only ischemic stroke but also long-term undesirable major cardiovascular events and systemic embolism. Bleeding complications after anticoagulant treatment and mortality risk is high [2,3].

Left ventricular wall motion score index (WMSI) is a simple alternative method to ejection fraction (EF) that is used to evaluate left ventricular systolic functions [4,5]. EF does not provide information about left ventricular regional systolic functions. After acute myocardial infarction (MI), EF may be normal even if myocardial damage is extensive [6]. WMSI consists of a scoring system that evaluates left ventricular segmental motion, with higher scores indicating a more severe change in wall motion [7]. Unlike EF after acute MI, it has been found to have prognostic importance in early risk classification as it provides information about regional systolic functions and is an independent predictor of death and hospitalization due to heart failure [8].

In this study consisting of patients treated mostly with primary percutaneous coronary intervention (PCI) as a reperfusion strategy, we tried to determine whether WMSI evaluated by transthoracic echocardiography (TTE) after acute STEMI is predictive of LVT.

## Materials and methods

Study design

In our prospective study, patients who were followed up and treated with a diagnosis of acute STEMI in the cardiology department of our tertiary heart disease center were evaluated sequentially. The risk factors, duration of pain, physical examination findings, and medications used by the patients included in the study group were obtained from health data records and file scans. Exclusion criteria were as follows: patients diagnosed with non-ST elevation acute coronary syndrome who are being followed up and treated; patients with limited echocardiography images or who do not attend control imaging appointments; those with previous LVT; patients who have previously used warfarin for any reason such as prosthetic valve, pulmonary embolism, or atrial fibrillation (AF); in-hospital mortality; acute stent thrombosis; patients who underwent revascularization with coronary bypass graft surgery; patients with noncritical coronary artery disease detected after coronary angiography; patients with advanced renal and hepatic insufficiency; and patients with advanced malignancy who received chemotherapy or radiotherapy for six months before the index procedure. With these criteria, 131 patients were included in the study. Our study was conducted according to the principles of the Declaration of Helsinki after obtaining written consent from each participant and with the approval of the local ethics committee. Our study was derived from the medical specialization thesis of one of our authors (D.K.).

Echocardiographic examination

All TTE examinations were performed based on images recorded with the Vivid 5 ECHO machine (General Electric Healthcare, Milwaukee, WI) four times: on the third and seventh days of hospitalization and at the first month and third months after discharge. Left ventricular (LV) diameters were measured using two-dimensional (2D) images in the parasternal long-axis window. Left ventricular EF (LVEF) was calculated using the modified Simpson method from apical two- and four-chamber views. LVT was defined as an echo-dense mass within the left ventricular cavity on 2D examination, with clearly visible borders throughout the cardiac cycle, visible from at least two separate windows, adherent to the hypokinetic or akinetic myocardium, but different in density from the endocardial tissue [9]. WMSI was calculated using the 16-segment model recommended by the American Society of Echocardiography [10]. Segments were examined separately using apical two- and four-chamber and parasternal long-axis images. Segmental wall movements scored as follows: normal = 1 point, hypokinetic = 2 points, akinetic = 3 points, and dyskinetic = 4 points. Wall motion scores of the segments were summed and divided by the number of segments examined, and WMSI was found. Pulsed wave (PW) and tissue Doppler recordings were used for left ventricular diastolic functions. Mitral inflow measurements were obtained by placing the sample volume at the point where the mitral valve tips opened maximum in diastole. In order, early diastolic flow peak velocity (E), late diastolic flow peak velocity (A), and E wave deceleration time (DT) were obtained. Tissue Doppler examinations were performed from the basal mitral annular regions of the lateral and septal walls. Systolic wave (Sm), early diastolic wave (Em), and late diastolic wave (Am) were measured from these regions with PW Doppler.

Measurement Consistency of the Sonographers in Our Study

Control ECHO images were tested on the measurements of patients made by the same sonographer and/or the same patient's measurements made by different sonographers (D.K. and Ş.Ç.) in our study. Inter-class correlation coefficient (ICCC) values were calculated for each of the echocardiography measurements obtained to evaluate the agreement of the sonographers (between D.K. and Ş.Ç.). The lowest ICCC value obtained was 0.77 (p=0.016), while the highest ICCC value was 0.91 (p<0.001).

Electrocardiographic examination

At hospital admission before coronary angiography, standard 12-lead surface electrocardiograms (ECGs) were evaluated. Subsequently, ECGs were assessed at the 60th minute after reperfusion was achieved and then taken daily as part of routine monitoring. We calculated the pathological Q wave presence, QT dispersion, and ST segment deviation scores. The diagnosis of STEMI was made in the presence of ≥1 mm ST elevation in at least two adjacent leads and reciprocal ST segment depression in the opposite leads. We defined the pathological Q wave as a Q wave duration of ≥0.02 seconds in V2-V3, ≥0.03 seconds in other derivations, and an amplitude of ≥1 mm or the presence of a QS complex. We measured the distance from the beginning of the QRS complex to the end of the T wave as the QT interval, and the difference in seconds between the maximum QT duration and the minimum QT duration was defined as QT dispersion (QTd). In leads with ST elevation, we calculated the ST segment deviation score by adding up the positive deviations in millimeters relative to the TP segment, which was determined as the isoelectric line.

Biochemical examination

Venous blood samples were taken to measure the patients' complete blood count and basic biochemical markers immediately after their admission to the intensive care unit, and the troponin results taken during the follow-ups were obtained by looking retrospectively at the hospital-based registration system.

Treatment

Patients who did not undergo primary PCI or thrombolytic treatment for any reason, did not receive reperfusion therapy during hospitalization, and were managed with anticoagulants and/or antithrombotics were considered to have received conservative treatment. Coronary angiography records of those who underwent primary PCI were examined, and the flow in the epicardial vessels before and after the procedure was evaluated as thrombolysis in myocardial infarction (TIMI) flow grade. In TIMI 0, there is no post-occlusion flow. In TIMI 1, the contrast material passes through the occlusion but cannot fill the distal coronary bed. In TIMI 2, contrast material fills the distal coronary bed, but the filling and washing rate is slower than in the normal artery. In TIMI 3, the rate of contrast material filling and washing out of the distal coronary bed is the same as in normal coronary arteries.

Post hoc power analysis

Post hoc power analysis was applied in our study. Based on the correlation coefficient of WMSI with the presence of LVT in the correlation point biserial model technique (effect size |ρ| = 0.6123724, type 1 error = 0.05), the sample size was reported as 131 and post hoc power was found to be 1.00.

Statistical analysis

The results are given as mean ± standard deviation and percentage values. We used the Kolmogorov-Smirnov test to determine whether the variables were homogeneously distributed. The significance of the statistical difference between the groups was evaluated with the chi-square test, Mann-Whitney U test, and independent sample t-test. We evaluated the relationship between LVT and demographic data using Pearson’s correlation analysis. Independent risk factors of LVT development were investigated using logistic regression analysis. We included the variables with p < 0.1 in univariate analyses in the modeling. Independent risk factors for the presence of LVT were evaluated using receiver operating characteristic (ROC) curve analysis. The Youden index determined the predictive value with the highest specificity and sensitivity. Statistical Package for the Social Sciences (SPSS) for Windows Version 21.0 (IBM Corp., Armonk, NY, USA) was used for statistical calculations. A p-value of <0.05 was considered statistically significant.

## Results

The study included 131 patients, of whom 84.7% were male. The patients were divided into two groups: those with thrombus detected as "thrombus (+)" and those with no thrombus detected as "thrombus (-)." We found the thrombus incidence to be 17.6% (23 patients). The mean age of the patients was 59.7 ± 11.7 years. No significant difference was observed between the groups in terms of demographic characteristics. The characteristic features of the patients are given in Table 1.

**Table 1:** Demographic, laboratory, electrocardiography, echocardiography, and angiography data of study patients and thrombus groups BMI, body mass index; DM, diabetes mellitus; CRF, chronic renal failure; MI, myocardial infarction; ACEI, angiotensin-converting enzyme inhibitor; PCI, percutaneous coronary intervention; TIMI, thrombolysis in myocardial infarction; CRP, C-reactive protein; WMSI, wall motion score index; EF, ejection fraction; DV, diastolic volume; SV, systolic volume; MR, mitral regurgitation

Variables	All patients (n = 131)	Thrombus (+) (n = 23)	Thrombus (-) (n = 108)	p-value
Male gender, n (%)	111 (84.7)	20 (86.9)	91 (84.2)	0.744
Age (year)	59.7 ± 11.7	62± 12.7	59.2 ± 11.5	0.313
BMI (kg/m²)	27.8 ± 4.6	28.1 ± 4.8	27.8 ± 4.5	0.809
Hypertension, n (%)	63 (48.1)	12 (52.1)	51 (47.2)	0.666
DM, n (%)	23 (17.5)	6 (26.1)	17 (15.7)	0.236
CRF, n (%)	3 (2.3)	1 (4.3)	2 (1.8)	0.467
Smoking, n (%)	64 (48.8)	14 (60.8)	50 (46.2)	0.204
Anterior MI, n (%)	78 (59.5)	20 (86.9)	55 (50.9)	<0.001
Non-anterior MI, n (%)	53 (40.4)	3 (13.1)	53 (49.1)	<0.001
Beta blocker, n (%)	116 (88.5)	22 (95.6)	94 (87)	0.239
ACEI, n (%)	112 (85.4)	22 (95.6)	90 (83.3)	0.128
Chest pain duration (hour)	3 (2–8)	5 (2–10)	3 (2–7)	0.500
Killip class ≥2, n (%)	8 (6.1)	4 (17.3)	4 (3.7)	0.011
Heart rate (beats/min)				
Baseline	78.4 ± 18.7	84.9 ± 23.6	77 ± 17.2	0.065
Discharge	75.0 ± 14.8	78.1 ± 15.1	74.4 ± 14.8	0.279
Treatment (%)				
Conservative	8 (6.1)	2 (8.7)	6 (5.5)	0.833
Fibrinolytic	7 (5.3)	1 (4.3)	6 (5.5)	
PCI	116 (88.6)	20 (87)	96 (89)	
Preangioplasty TIMI flow grade ≤1, n (%)	76 (58)	18 (78.2)	58 (53.7)	0.001
Postangioplasty TIMI flow grade ≤1	5 (3.8)	1 (4.3)	4 (3.7)	0.295
Door-to-balloon time (minutes)	43.4 ± 12.6	45.6 ± 12.4	42.9 ± 13.1	0.163
Glucose (mg/dL)	89.3 ± 21.9	91.4 ± 21.6	93.3 ± 20.5	0.247
Creatinine (mg/dL)	0.91 ± 0.13	0.96 ± 0.22	0.86 ± 0.24	0.195
Hemoglobin (g/dL)	11.7 ± 1.6	10.6 ± 1.3	12.5 ± 1.7	0.082
Platelet (10^3^/uL)	216.65 ± 59.88	194.47 ± 48.65	221.37 ± 61.17	0.050
Troponin (ng/mL)	36.0 ± 18.3	39.2 ± 18.3	35.3 ± 18.3	0.359
CRP (mg/dL)	3 (2–6)	4 (3–7)	3 (2–6)	0.062
Pathological Q wave (number of derivations)				
Baseline	1.3 ± 1.4	1.87 ± 1.51	1.21 ± 1.47	0.056
Discharge	2.1 ± 1.5	3.17 ± 1.37	1.93 ± 1.45	<0.001
QT dispersion				
Baseline	0.09 ± 0.02	0.07 ± 0.02	0.10 ± 0.23	0.572
Discharge	0.08 ± 0.02	0.06 ± 0.02	0.09 ± 0.02	0.617
ST deviation score				
Baseline	11.7 ± 7.8	15.8 ± 9.2	10.8 ± 7.2	0.005
Discharge	4.0 ± 4.2	6.6 ± 4.8	3.5 ± 3.9	0.001
WMSI	1.5 ± 0.4	2.1 ± 0.44	1.40 ± 0.31	<0.001
EF (%)	45.2 ± 11.7	32.2 ± 8.4	47.9 ± 10.3	<0.001
DV (mL)	97.1 ± 30.6	116.1 ± 43.9	93.1 ± 25.5	0.001
SV (mL)	54.1 ± 25.5	79.5 ± 36.1	48.7 ± 18.8	<0.001
Diastolic dysfunction, n (%)	80 (61.1)	12 (52.1)	68 (62.9)	0.335
MR ≥ moderate, n (%)	45 (34.4)	12 (52.1)	33 (30.5)	0.047
Aneurysm, n (%)	12 (9.2)	10 (43.4)	2 (1.8)	<0.001

While the anterior STEMI rate was 86.9% in the thrombus (+) group, it was 50.9% in the thrombus (-) patient group (p<0.001). The thrombus (+) group was significantly more symptomatic (Killip class ≥2) than the thrombus (-) group. Primary PCI was performed in 88.6% of the patients included in the study. Fibrinolytic treatment was given to 5.3% of the patients, while conservative treatment was given to 6.1%. The treatments applied were similar between groups. In our procedural evaluation, pre-angioplasty TIMI flow grade ≤1 was observed in 78.2% of the thrombus (+) group, while it was in 53.7% of the thrombus (-) group (p=0.001). Post-angioplasty TIMI flow grade did not create a significant difference between groups. We detected no significant difference between the groups in laboratory tests. While the number of ECG leads with pathological Q was similar between the groups at admission, it was observed to be significantly higher in the thrombus (+) group at discharge (3.17 ± 1.37 vs. 1.93 ± 1.45; p<0.001). ST segment deviation score was significantly higher in the thrombus (+) group at admission and discharge. While WMSI was 2.1 ± 0.44 in the thrombus (+) group, it was 1.40 ± 0.31 in the thrombus (-) group (p<0.001). EF was measured as 32.2 ± 8.4% in the thrombus (+) group and 47.9 ± 10.3% in the thrombus (-) group (p<0.001). End-diastolic and end-systolic volumes, mitral regurgitation, and aneurysm rates were found to be significantly higher in the thrombus (+) patient group.

We evaluated the correlation between the presence of LVT and other variables by Pearson’s correlation analysis (Table 2). Anterior MI, Killip class ≥2, number of pathological Q leads (at discharge), ST segment deviation score (at admission and discharge), C-reactive protein (CRP), end-diastolic volume, and mitral regurgitation were found to have a significant positive correlation with thrombus. WMSI was identified as the highest variable, with a correlation coefficient of 0.613 (p<0.001). This was followed by the presence of the aneurysm (r: 0.549; p<0.001) and end-systolic volume (r:0.461; p<0.001). EF was found to have a significant negative relationship with the presence of the thrombus (r: -0.514; p<0.001).

**Table 2:** Correlation between the presence of thrombus and other demographic variables BMI, body mass index; STEMI, ST-segment elevation myocardial infarction; DM, diabetes mellitus; CRF, chronic renal failure; WMSI, wall motion score index; MPV, mean platelet volume; CRP, C-reactive protein, MR, mitral regurgitation

Variables	Pearson Correlation	p-value
Age	0.089	0.313
Gender	0.029	0.746
BMI	0.021	0.809
Anterior STEMI	0.380	<0.001
Hypertension	0.038	0.669
DM	0.103	0.240
CRF	0.063	0.471
Smoking	0.111	0.207
Killip class ≥2	0.233	0.007
Baseline heart rate	0.162	0.065
Discharge heart rate	0.095	0.279
Baseline pathological Q	0.168	0.056
Discharge pathological Q	0.316	<0.001
Baseline QT dispersion	-0.050	0.572
Discharge QT dispersion	-0.044	0.617
Baseline ST deviation score	0.242	0.005
Discharge ST deviation score	0.282	0.001
WMSI	0.613	<0.001
Aneurysm	0.549	<0.001
Platelet	-0.172	0.050
MPV	0.047	0.595
Troponin	0.081	0.359
CRP	0.240	0.006
Treatment	-0.037	0.672
Pain duration	-0.059	0.500
Diastolic volume	0.268	0.001
Systolic volume	0.461	<0.001
Ejection fraction	-0.514	<0.001
Diastolic dysfunction	-0.084	0.339
MR ≥ moderate	0.173	0.048

We performed the univariate and multivariate logistic regression analysis to determine independent risk factors for the presence of LVT (Table 3). As a result of multivariate analysis, anterior MI, number of leads with pathological Q wave at discharge, WMSI, aneurysm detection, and EF were identified as independent risk factors for the presence of LVT. WMSI was found to be the most potent risk factor with an odds ratio (OR) of 7.971 (95% confidence interval [CI]: 2.377-14.517; p=0.012). This was followed by anterior MI (OR: 4.266; 95% CI: 2.012-7.052; p<0.001) and the number of leads with pathological Q wave at discharge (OR: 3.651; 95% CI: 1.385-9.622; p=0.009).

**Table 3:** Independent predictors of thrombus in logistic regression analysis OR, odds ratio; CI, confidence interval; BMI, body mass index; MI, myocardial infarction; DM, diabetes mellitus; CRF, chronic renal failure; WMSI, wall motion score index; CRP, C-reactive protein, EF, ejection fraction, MR, mitral regurgitation

Variables	Univariate analysis			Multivariate analysis		
	OR	95% CI	p-value	OR	95% CI	p-value
Age	1.020	0.982–1.060	0.311			
Gender	1.245	0.333–4.659	0.744			
BMI	1.012	0.919–1.114	0.807			
Anterior MI	4.486	2.602–7.605	<0.001	4.266	2.012–7.052	<0.001
Hypertension	1.219	0.495–3.002	0.666			
DM	1.889	0.651–5.481	0.242			
CRF	2.409	0.209–27.750	0.481			
Smoking	1.804	0.720–4.523	0.208			
Killip class ≥2	3.649	0.945–23.320	0.056	0.956	0.048–18.98	0.976
Baseline pathological Q	1.320	0.988–1.763	0.060	1.242	0.969–2.291	0.115
Discharge pathological Q	1.849	1.293–2.644	0.001	3.651	1.385–9.622	0.009
Baseline ST deviation score	1.076	1.019–1.136	0.008	1.084	0.967–1.215	0.166
Discharge ST deviation score	1.158	1.049–1.277	0.003	0.865	0.665–1.125	0.279
WMSI	27.047	2.630–278.12	<0.001	7.971	2.377–14.517	0.012
Aneurysm	4.076	2.038–20.678	<0.001	2.089	1.547–7.974	0.009
CRP	1.131	1.026–1.246	0.013	1.127	0.909–1.396	0.275
Diastolic volume	1.022	1.007–1.036	0.003	0.865	0.617–1.214	0.402
Systolic volume	1.047	1.023–1.071	<0.001	1.230	0.735–2.057	0.430
EF	1.189	1.104–1.280	<0.001	1.129	1.037–1.856	0.006
MR ≥ moderate	2.479	0.993–6.189	0.052	0.989	0.936–1.738	0.161

**Figure 1:**
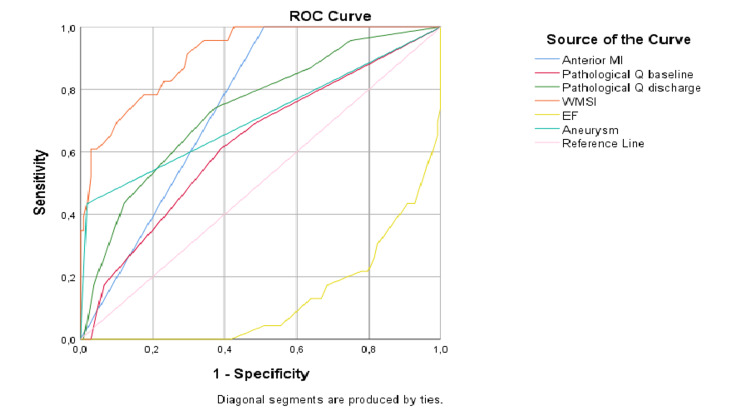
Receiver-operator curves for left ventricular thrombus predictors. MI, myocardial infarction; WMSI, wall motion score index; EF, ejection fraction

The variables we defined as independent risk factors for the presence of LVT were evaluated in ROC analysis to compare their diagnostic power in detecting the presence of thrombus (Figure 1). The variable with the highest diagnostic power for the presence of thrombus was determined to be WMSI. The area under the curve (AUC) for WMSI was found to be 0.910 (95% CI: 0.852-0.968) (Table 4). A WMSI cut-off of 1.56 identified LV thrombus with 91% sensitivity and 70% specificity (Youden index: 0.617).

**Table 4:** Receiver-operator curve results for left ventricular thrombus predictors. MI, myocardial infarction; WMSI, wall motion score index; EF, ejection fraction

Variables	Area	p-value	95% confidence interval	
			Lower bound	Upper bound
Anterior MI	0.745	<0.001	0.659	0.832
Pathological Q baseline	0.527	0.096	0.400	0.655
Pathological Q discharge	0.737	<0.001	0.624	0.849
WMSI	0.910	<0.001	0.852	0.968
EF	0.872	<0.001	0.800	0.945
Aneurysm	0.708	0.002	0.570	0.846

## Discussion

The most important findings in this study consisting of real-life data are as follows. The incidence of LVT after acute STEMI is still high despite aggressive reperfusion strategy and antiplatelet therapy. Anterior STEMI, pathological Q wave count at discharge, presence of aneurysm, WMSI and low LVEF are independent predictors of LVT. Among the independent predictors, the variable with the highest diagnostic power for LVT is WMSI.

While the incidence of LVT varies between 17% and 24% in studies conducted before primary PCI, it appears to be between 27% and 39% in anterior MI [11-14]. In a meta-analysis including 19 studies using TTE in the primary PCI period, the incidence was 2.7%, while it was 9.1% in anterior MI [15]. When cardiac magnetic resonance imaging (MRI) is used for diagnosis, the incidence is even higher (6.3% in all STEMI, 12.2% in anterior MI) [1]. Compared to MRI, the sensitivity of TTE in diagnosing LVT is lower (35% vs. 64%) [16]. In our study, the incidence of LVT detected by TTE was approximately 17%, while it was 25% in anterior MI. Compared to primary PCI period data, the incidence of LVT appears to be higher in our study. Even if there was no statistically significant difference between the two groups, the presence of patients who did not receive reperfusion therapy, the longer door balloon time, and the hospital admission time after the onset of symptoms in the group with thrombus detection may have facilitated thrombus formation after MI. In addition, although post-PCI TIMI flow was sufficient in most of those who underwent primary PCI, the pathological Q wave count at discharge was significantly higher in the LVT group. The pathological Q wave after reperfusion indicates that the infarct area is larger and myocardial perfusion and LV functions are worse [17]. All these factors mentioned above can be considered among the possible reasons why the incidence of LVT was high in our study.

In our study, the cases in which thrombus was detected predominantly consisted of anterior STEMI patients. This finding is consistent with previous studies showing that most thrombosis occurs after anterior STEMI [18,19]. We also found low LVEF [20] and aneurysm formation [16,21] to be associated with thrombus formation, similar to previous studies. It is known that the majority of left ventricular aneurysms after MI occur after total occlusion of the left anterior descending artery and that EF decreases after aneurysm formation. The slowdown of blood flow in the aneurysmatic segments and the contact of procoagulant fibrous tissue with blood increase the possibility of thrombus formation in these regions [22].

The most important finding in our study is WMSI, which we found to be the variable with the highest diagnostic power for LVT among independent risk factors. It is expected that complications will occur at numerically higher values. Higher WMSI values indicate worse affected left ventricular area and segment functions. As segmental systolic movements decrease, WMSI increases in numerical value. In scoring, akinetic and dyskinetic segments receive higher scores, and it is already known that the probability of thrombus formation is higher in these regions. Few studies demonstrate the relationship between LVT and WMSI after MI. In two studies conducted with fibrinolytics, the number of cases was relatively low [23,24].

In the study conducted by Nesković et al. [23], reperfusion treatment was not applied to half of the cases, and streptokinase was used as fibrinolytic in the other half. In this study, TIMI 3 flow was observed in only 63% of the patients during coronary angiography performed for pre-discharge control. We could not find any other study in the literature showing the relationship between WMSI and thrombus in a group including patients, most of whom underwent primary PCI. A study including a few patients who underwent primary PCI showed a relationship between WMSI height and thrombus [25]. However, in this study, fibrinolytic treatment was performed in most cases in the first stage, and rescue PCI was applied to those in whom reperfusion could not be achieved. The number of patients who underwent primary PCI in this study is relatively low compared to our study. In studies involving acute MI patients, WMSI was found to be superior to EF in predicting death, heart failure [26,27], and hospitalization due to congestion [8], and was shown to be a stronger prognostic predictor than EF even when myocardial damage was less [28]. Among all the methods used to measure left ventricular function (compared to EF measured by standard ECHO, contrast ECHO, or single-photon emission computed tomography), the highest relationship with global strain was observed with WMSI [29]. Data on prognosis and complications after MI are gradually increasing with WMSI, which is based on the evaluation of segmental systolic movements after acute MI. Our study, which shows the relationship between LVT and WMSI after MI, consists almost entirely of patients who underwent primary PCI and has no other example in this sense, and this will make a significant contribution to the literature.

Limitations

Our study has some limitations. First of all, our small sample size is a significant limitation. Another area for improvement is that our average time from symptom onset to diagnosis is high. This situation is because transportation from rural areas to our reference hospital center depends on a stepped referral chain. WMSI measured by 2D TTE is a semiquantitative, experience-dependent, subjective assessment of myocardial movements that examines segment movements. WMSI measurements were standardized by testing consistency among the sonographers performing the evaluation and trying to keep them at optimal levels as much as possible. Large-scale studies using MRI and strain echocardiography, which provide a more objective evaluation of LV functions and segment movements, will be able to provide more advanced information. However, the costs of these studies and their applicability in clinical practice should also be considered.

## Conclusions

In the era of primary PCI, the incidence of LVT after acute STEMI is still substantial. WMSI is the variable with the highest diagnostic power detected in this study in predicting LVT. It should be considered an essential parameter, among other variables, in determining patients who may develop complications after MI and who need aggressive treatment and close monitoring.
